# Spatial variations impact the soil fungal communities of *Larix gmelinii* forests in Northeast China

**DOI:** 10.3389/fpls.2024.1408272

**Published:** 2024-05-24

**Authors:** Wen Zhao, Kaichuan Huang, Reyila Mumin, Junning Li, Yifei Sun, Baokai Cui

**Affiliations:** State Key Laboratory of Efficient Production of Forest Resources, School of Ecology and Nature Conservation, Beijing Forestry University, Beijing, China

**Keywords:** climatic factors, fungal community assembly, *Larix gmelinii* forest, physicochemical properties, soil fungi

## Abstract

Soil fungi play a critical role in the biogeochemical cycles of forest ecosystems. *Larix gmelinii* is a strong and important timber tree species, which forms close associations with a wide range of soil fungi. However, the temporal-spatial disparity effects on the assembly of soil fungal communities in *L. gmelinii* forests are poorly understood. To address these questions, a total of 120 samples, including 60 bulk soil and 60 root samples, were collected from Aershan and Genhe in July (summer) and October (autumn)2021. We obtained 7,788 operational taxonomic units (OTUs) after merging, filtering, and rarefying using high-throughput sequencing. The dominant phyla are Basidiomycota, Ascomycota, Mortierellomycota, and Mucoromycota. There were 13 dominant families, among which the families with average relative abundance more than 5% included Thelephoraceae, Mortierellaceae, Archaeorhizomycoaceae, and Inocybaceae. In the functional guilds, symbiotrophic fungi had a relative advantage in the identified functions, and the relative abundances of pathotrophic and saprotrophic fungi varied significantly between sites. There were 12 families differentially expressed across compartments, 10 families differentially expressed between seasons, and 69 families were differentially expressed between sites. The variation in alpha diversity in the bulk soil was greater than that in the rhizosphere soil. Among the three parts (compartment, season, and site), the site had a crucial effect on the beta diversity of the fungal community. Deterministic processes dominated fungal community assembly in Genhe, whereas stochastic processes dominated in Aershan. Soil physicochemical properties and climatic factors significantly affected fungal community structure, among which soil total nitrogen and pH had the greatest effect. This study highlights that spatial variations play a vital role in the structure and assembly of soil fungal communities in *L. gmelinii* forests, which is of great significance for us in maintaining the health of the forests.

## 1 Introduction

As an important component of soil microorganisms, soil fungi play a vital role in the cycling of carbon, nitrogen, and phosphorus in the ecosystem (Lladó et al., 2018) and promote the biogeochemical cycle of the earth (Crowther et al., 2019; Jiang et al., 2021). Among these soil fungi, mycorrhizal fungi established mutualistic interaction with their hosts (Zhong et al., 2021), saprophytic fungi carried out material and energy cycling in soil (Setälä and McLean, 2004), and pathogenic fungi were partly transferring in soil and causing disease (Maron et al., 2011). The closest connection between soil fungi and plants occurs in the rhizosphere, which is due to the root exudates of the plant and nutrient availability of the soil (Li et al., 2016; Praeg and Illmer, 2020). At the same time, plants also choose relevant fungi, so vegetation types have a significant impact on the fungal community in rhizosphere soil (Berendsen et al., 2012; Liu et al., 2023).

*Larix gmelinii* is a deciduous tree of the Pinaceae family with rich timber accumulation and extreme cold tolerance (Kajimoto et al., 1999; Sun et al., 2007). Northeast China is mainly located in the Greater Khingan Mountains, which are important forest resources. *Larix gmelinii* is an ectomycorrhizal tree species (Ouimette et al., 2013; Miyamoto et al., 2021; Meng et al., 2023). Numerous fungi attach to their host for nutrient uptake (Ishida et al., 2007; Orwin et al., 2011). It can also help plants absorb nutrients (Kuang et al., 2021) and maintain health (Berendsen et al., 2012). Complex plant-associated fungal communities are directly influenced by organic compounds secreted from plant roots (Broeckling et al., 2008).

Fungal communities in the forest soil exhibited strong temporal and spatial differences. Previous studies showed that ectomycorrhizal fungi play a significant role in plant growth season, whereas saprophytic fungi evidently increase during the cold season (Voříšková et al., 2014; Santalahti et al., 2016). Seasonal changes in temperature and precipitation are also responsible for variations in soil fungal communities (Tedersoo et al., 2014; Zhang et al., 2018). In our previous study, temperature and precipitation were found to be the most important factors affecting fungal diversity with seasonal variations (Zhao et al., 2023). As for spatial differences, soil fungal communities have different structures between geographic sites, and their physicochemical properties directly constitute different fungal living environments, such as nitrogen (Zhou et al., 2016), pH (Zhang et al., 2016), and moisture (Zumsteg et al., 2013). Our previous study showed that differences in temperature and precipitation are also the main driving factors for differences in fungal communities at large regional scales (Wang et al., 2022). Even at a small local scale, there are significant differences in soil fungal communities among different vegetation types (Huang et al., 2023).

Community assembly, which is the balance between fungal community activity and environmental filtration, is categorized into deterministic and stochastic processes. Deterministic processes involve biotic and abiotic factors, including interspecific interactions such as competition, predation, symbiosis, and environmental filtering. Stochastic processes are understood as the drift, migration, speciation, and species extinction of microorganisms (Nemergut et al., 2013). When the environment limited the life of fungi, deterministic processes were the main factors affecting communities. Therefore, the communities dominated by deterministic processes were more unstable. In contrast, the communities dominated by stochastic processes were resistant to environmental change (Powell et al., 2015). It is difficult to understand the key factors that affect soil fungal community assembly processes, because we cannot directly measure an indicator to gauge the process. Ecologists have obtained a deeper and more scientific understanding of the community assembly process and shown that the stochastic and deterministic processes can be strongly influenced by selection, dispersal, diversification, and drift (Vellend, 2010; Stegen et al., 2012), and compute a value through a framework to delineate the processes (Stegen et al., 2013). Zhou and Ning (2017) suggested that one of the main goals of microbial ecology is to project future scenarios of microbial community structure and functions in a changing environment.

In this study, we aimed to determine the differences in rhizosphere and bulk soil fungal communities with different temporal and spatial variations and to determine the relationship between the fungal community and environmental variables. Considering the long winters and frozen soil in Frigid-Temperate Zones, and combining with the annual variation in temperature and precipitation, we finally chose July and October to represent the seasons (summer and autumn). We selected two representative sites in the main distribution of *L. gmelinii* in the Greater Khingan Mountains and collected 120 rhizosphere and bulk soil samples for analysis and exploration in July and October of 2021. Furthermore, we identified the main taxa and their functional guilds, summarized their variations, and calculated the community assembly processes and the main factors that caused these results. This was a multifaceted systematic analysis to explore the temporal-spatial patterns and driving factors of the soil fungal community in *L. gmelinii* forest.

## 2 Materials and methods

### 2.1 Study sites

**Figure 1:**
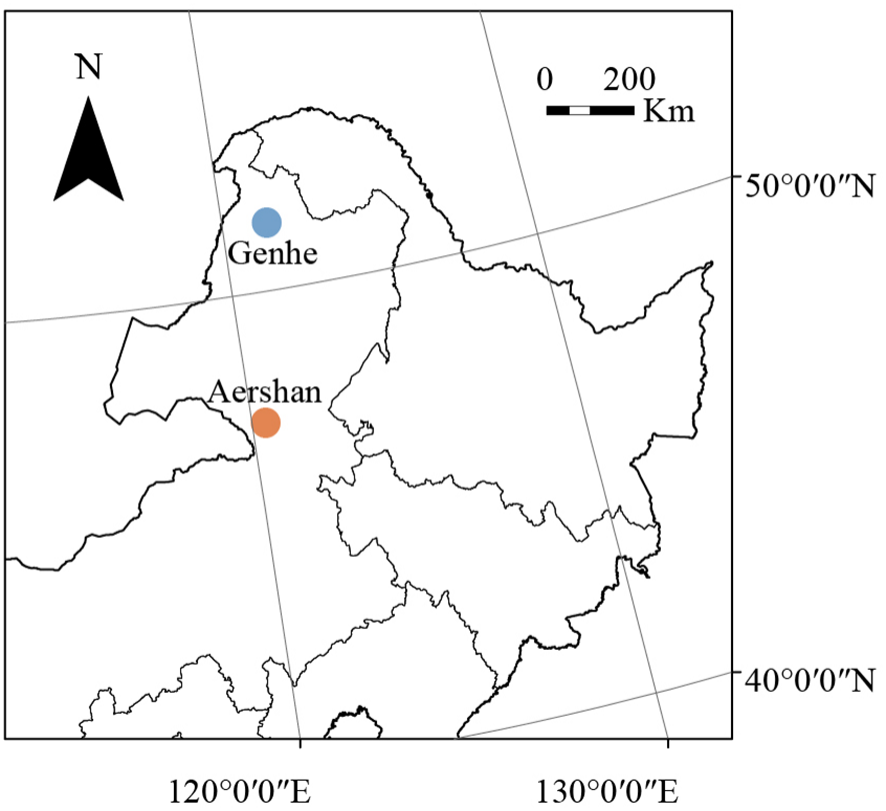
Distribution of the two forests.

The present study was conducted in two typical areas of *L. gmelinii* forests in the Greater Khingan Mountains, China (**Figure 1**, **Table 1**). One study area was located in the Aershan National Forest Park, Aershan (47°15′17″N, 120°17′34″E, shortened to Aershan), the southernmost area of *L. gmelinii* growing in China. The other was located in the Jinlin Forest Farm, Genhe (51°21′51″N, 121°20′31″E, shortened to Genhe), which is the core area for the distribution of *L. gmelinii*. The study area is located in a high-latitude permafrost distribution area in China (Ding et al., 2019). The winters in the study areas were long and cold, the summers were short and rainy, and spring and autumn were cool with less precipitation (Liu et al., 2020). The dominant tree species in these areas were *L. gmelinii* and *Betula platyphylla*, with a forest coverage rate of 80%. The zonal soil at both sites is Retisol (IUSS Working Group, 2015). The soil in Aershan is a typical black soil that is soft, moist, and nutritious, whereas the soil in Genhe is rich in gravel. A shovel was used to turn over the stones and obtain the soil in the stone cracks.

**Table 1:** Localities, climates and forest conditions of the sampling sites.

Site	Latitude, longitude	MAT (°C)	MAP (mm)	Altitude (m)	Average tree height (m)	DBH (cm)	Tree age (year)	Understory
Aershan	47°15′17″N, 120°17′34″E	−2.87	459	1065	14	18	40	Grass
Genhe	51°21′51″N, 121°20′31″E	−5.11	507	915	12	15	60	*Ledum palustre*

MAT, Mean Annual Temperature; MAP, Mean Annual Precipitation; DBH, diameter at breast height.

### 2.2 Collection of bulk and rhizosphere soil

The rhizosphere and bulk soil samples were collected in July and October 2021, representing summer and autumn, respectively. At each sampling site, we established three 20 m × 20 m plots at least 100 m apart and collected five soil samples from five points (four corners and the center) as independent samples. Roots were extracted by digging the trees near the points to collect rhizosphere soil. In total, 120 samples (5 independent replicates× 3 plots × 2 compartments × 2 sites × 2 seasons) were used for DNA extraction. Additionally, 60 bulk soil samples were used to determine the soil physicochemical properties. Specifically, bulk soil from 0 to –20 cm was obtained, sifted through a 2 mm mesh sieve, mixed, and then packed into centrifuge tubes and sealed bags. The roots were stored in sealed bags. The samples in centrifuge tubes were stored with dry ice during the sampling process and transported to the laboratory stored at −80°C. The roots were stored with ice in the field and then transported to the laboratory stored at 4°C for the rhizosphere soil. The bulk soil was stored in sealed bags and transported at room temperature.

Rhizosphere soil was then separated from roots by shaking root and soil in 25 mL 0.1 M sterile phosphate buffer (7.1 g Na_2_HPO_4_ and 4.4 g NaH_2_PO_4_·H_2_O were added to 820 mL deionized water; pH 7.0) in a 50 mL plastic conical centrifuge tube at 67 g for 5 min. The soil suspension was centrifuged at 4000 g for 15 min, and the obtained soil pellet was regarded as the rhizosphere soil and stored at −80°C until DNA extraction (Liu et al., 2021).

### 2.3 Environmental variables

To determine soil properties, we followed the method described by Bao (2000). The soil in the sealed bags was dried naturally to determine the following physicochemical properties: soil organic carbon (SOC) was determined by the potassium dichromate volumetric method (Li et al., 2020), available phosphorus (AP) was determined by molybdenum antimony resistance colorimetry (Cai et al., 2018), pH was determined by the potential method (Li et al., 2020), total nitrogen (TN) was determined by Kjeldahl method (Shi et al., 2014), and the cation exchange capacity (CEC) was determined by the ammonium acetate exchange method (Liang et al., 2015).

Historical monthly weather data were obtained from WorldClim (https://www.worldclim.org/data/monthlywth.html#; accessed July 20, 2023), including average minimum temperature (°C), average maximum temperature (°C), and total precipitation (mm) in July and October 2021. The mean annual temperature and precipitation for 1970–2000 were obtained from WorldClim.

### 2.4 Soil DNA extraction and PCR amplification

DNA was extracted using the DNeasy PowerSoil Pro Kit (Qiagen, Frankfurt, Germany). A NanoDrop NC2000 spectrophotometer (Thermo Fisher Scientific, Waltham, MA, USA) was used to quantify the DNA, and the quality of the DNA was detected using 1.2% agarose gel electrophoresis. The ITS1 region of fungi was amplified, and the primers were ITS5F (5-GGAAGTAAAGTAAAAGTCGTAAAGG-3) and ITS2R (5-GCTGCGTTCTTCATCGATGC-3) (White et al., 1990). PCR system (20 μL): 2 μL (2.5 mM) of dNTP. Of note, 1 μL (10 µM) forward primers and 1 μL (10 µM) reverse primers, 2 μL template DNA, 10 μL ddH2O, and 4 μL Fast pfu DNA polymerases. Circulating system: 95°C for 2 min; 30 cycles at 95°C for 30 seconds, 55°C for 30 seconds, and 72°C for 30 seconds; 72°C for 5 min. PCR amplification was performed using an Applied Biosystems 2720 Thermal Cycler (Thermo Fisher Scientific, Waltham, MA, USA). The Quant-iT PicoGreen dsDNA assay was performed using a Microplate Reader FLx800 (BioTek, Burlington, Vermont, USA). PCR amplicons were purified and recovered by adding Vazyme VAHTSTM DNA Clean Beads (Vazyme, Nanjing, China), quantified with the fluorescent reagent of Quant-iT PicoGreen dsDNA assay kit (Invitrogen, Carlsbad, CA, USA), and then mixed in proportion to the sequencing amount. Pair-end 2 × 250 bp sequencing was performed using the Illumina MiSeq platform with a NovaSeq 6000 SP reagent kit at Shanghai Personal Biotechnology Co., Ltd. (Shanghai, China). All raw sequencing data from this study have been deposited in the NCBI database with the Short Read Archive (SRA) accession number PRJNA1037725.

### 2.5 Sequence analysis

The early sequence processing in this experiment was based on QIIME 2–2021.2 (Bolyen et al., 2019). A Demux plug-in was used to split the samples. The DADA2 plug-in was used to perform quality control, such as filtering and noise removal; the positions at which the forward and reverse read sequences should be truncated due to a decrease in quality were 222 and 232, respectively, and the minimum and maximum sequence lengths were 230 and 438, respectively. The Vsearch plug-in was used to cluster the sequences into operational taxonomic units (OTUs) according to the 97% similarity principle. The Phylogeny plug-in was used to generate the phylogenetic trees. According to the fungus UNITE v.8.2 database (Nilsson et al., 2019), we used the Feature Classifier plug-in to annotate the species. Functional guilds and trophic modes were obtained from the FUNGuild database (Nguyen et al., 2016).

ITS is not a conserved region, making the constructed phylogenetic trees unstable. In this case, a hybrid tree based on the Silva 138 18S database and Unite 8.2 fungal dynamic database was constructed using q2-ghost-tree (Fouquier et al., 2016). The results of species composition and diversity analysis based on the OTU selected according to the ghost tree were basically consistent with those of the ITS tree, and the community assembly processes of the two phylogenetic trees were consistent. However, compared to phylogenetic trees based on ITS primers, the minimum frequency of samples decreased from 39,602 to 9,499, resulting in the loss of 76% of OTUs. Therefore, we decided to construct the ITS phylogenetic tree in this study.

### 2.6 Statistical analysis

The data processing part was mainly completed with R 4.1.2 (R Core Team, 2022) and SPSS 26.0 (SPSS Inc., Chicago, IL, USA). The vegan package provides tools for describing the community ecology. First, we used the rarefy command of the vegan package to standardize the OTU table according to the minimum sequence number of samples and constructed the rarefaction curve using the rare-curve command. Counts of different taxonomic categories were obtained using the Phyloseq package. Then we grouped taxa and functions which was not identified or whose average relative abundance was less than 1% into “others,” and defined dominant taxa as which a relative abundance more than 1%. The MicrobiotaProcess package was used to obtain alpha diversity indices. For alpha diversity analysis, the Shannon diversity index, Chao1 richness index, and Pielou evenness index were selected for one-way analysis of variance (ANOVA) and Tukey’s honest significant difference (HSD) test at *P* < 0.05 to compare species diversity. Beta diversity was tested by Principal coordinate analysis (PCoA) based on the OTU level, and the differences between fungal communities were tested by Permutational multivariate analysis of variance (PERMANOVA) of 999 permutations. Differential expression analysis was performed using the DESeq2 package based on the family level except for unassigned families. Comparisons were performed in four groups for each part to avoid the influence of other parts, and differentially expressed families (P < 0.05) were categorized as up regulated (log2FoldChange > 2) or down regulated (log2FoldChange < −2). A family that was up regulated or down regulated in two or more groups in each part was defined as differentially expressed in this part. We selected the null model described by Stegen et al. (2013) to classify community assembly processes. We calculated the β-nearest taxon index (βNTI) and modified Raup-Crick index (RC_Bray_) using iCAMP package. βNTI values < −2 (homogeneous selection) or > +2 (heterogeneous selection) indicated deterministic processes; −2 < βNTI values <+2 indicated stochastic processes including homogenizing dispersal (RC_Bray_ < −0.95), dispersal limitation (RC_Bray_ > 0.95) and undominated (−0.95 < RC_Bray_ < 0.95). The Mantel test was performed on community structure and environmental variables. A heatmap was used to show Spearman’s correlation between the relative abundance of dominant phyla and environmental variables. The data were visualized using the ggplot2 package. All analyses of variance (ANOVA) were calculated using SPSS, with *P* < 0.05 considered statistically significant.

## 3 Results

### 3.1 Soil properties and climatic factors

We only analyzed the soil properties in bulk soil, as we can only extract a small amount of rhizosphere soil for DNA extraction. There were significant differences in the physicochemical properties of the soil among the different variables (**Table 2**). Soil total nitrogen (TN), SOC, pH, and CEC in Aershan were significantly higher than those in Genhe. Available phosphorus (AP) in Genhe was significantly higher than that in Aershan. Between the two seasons, there were no significant differences in TN, SOC, and CEC in Aershan, and no significant difference in pH in Genhe. In summary, compared with the season, the differences in soil physicochemical properties between the two sites were greater.

**Table 2:** Soil properties compared by ANOVA (mean values ± S.E.s) and monthly average climate factors in 2021.

Environmental factor	Factor	BAJ	BAO	BGJ	BGO
Soil property	TN (g/kg)	5.79 ± 0.12a	5.77 ± 0.16a	2.61 ± 0.21b	1.87 ± 0.26c
	SOC (g/kg)	72.21 ± 1.18a	75.56 ± 2.48a	71.08 ± 7.22ab	49.97 ± 8.45b
	AP (mg/kg)	10.59 ± 0.87bc	10.04 ± 0.83c	16.43 ± 2.25ab	18.32 ± 2.02a
	pH	5.02 ± 0.05b	5.81 ± 0.05a	4.33 ± 0.07c	4.38 ± 0.08c
	CEC (cmol(+)/kg)	46.72 ± 0.39a	46 ± 0.57a	40.08 ± 1.42a	28.69 ± 3.76b
Climate factor	Tmax (°C)	23	7	22	5
	Tmin (°C)	12	−7	10	−10
	Prec (mm)	232.30	19.10	191	19.20

BAJ, bulk soil in Aershan, July; BAO, bulk soil in Aershan, October; BGJ, bulk soil in Genhe, July; BGO, bulk soil in Genhe, October; TN, total nitrogen; SOC, soil organic carbon; AP, available phosphorus; CEC: cation exchange capacity; Tmax, the monthly minimum temperature; Tmin, the monthly minimum temperature; Prec: mean monthly precipitation. Different letters indicate significant differences among different season tested by one-way ANOVA (P < 0.05).

### 3.2 Fungal community composition and functional guilds

**Figure 2:**
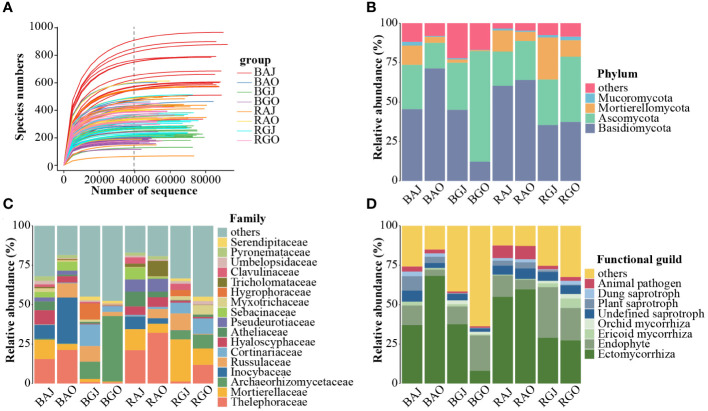
Fungal community structure in different compartments, seasons, and sites. **(A)** The rarefaction curve of the total 120 samples. Fungal community composition at phylum **(B)** and family **(C)** levels, and the functional guilds **(D)** of the community. In Figure **(D)**, red represents pathotroph, a series of blue represents saprotroph, a series of green represents symbiotroph. A, Aershan; G, Genhe; J, July; O: October; B, Bulk; R, Rhizosphere. BAJ, bulk soil in Aershan, July; BAO, bulk soil in Aershan, October; BGJ, bulk soil in Genhe, July; BGO, bulk soil in Genhe, October; RAJ, rhizosphere soil in Aershan, July; RAO, rhizosphere soil in Aershan, October; RGJ, rhizosphere soil in Genhe, July; RGO, rhizosphere soil in Genhe, October.

In total, 7,593,884 quality-filtered sequences were obtained from the 120 samples. After rarefying 39,602 sequences per sample (**Figure 2A**), 4,752,240 unique sequences were clustered into 7,788 operational taxonomic units (OTUs) with 97% identity. These OTUs belonged to 17 phyla, 60 classes, 138 orders, 362 families, and 864 genera.

We found that the differences in fungal composition were significant (**Figures 2B, C**), and there were also significant differences in functional guilds between the groups (**Figure 2D**). Basidiomycota, Ascomycota, Mortierellomycota, and Mucoromycota were the dominant phyla (average relative abundance > 1%). There were 13 dominant families, among which the families with a relative abundance more than 5% included Thelephoraceae, Mortierellaceae, Archaeorhizomycoaceae, and Inocybaceae. In terms of functional guilds, symbiotrophic fungi had an absolute advantage in the identified functions and the relative abundances of pathotrophic and saprotrophic fungi in Aershan were significantly higher than those in Genhe.

**Figure 3:**
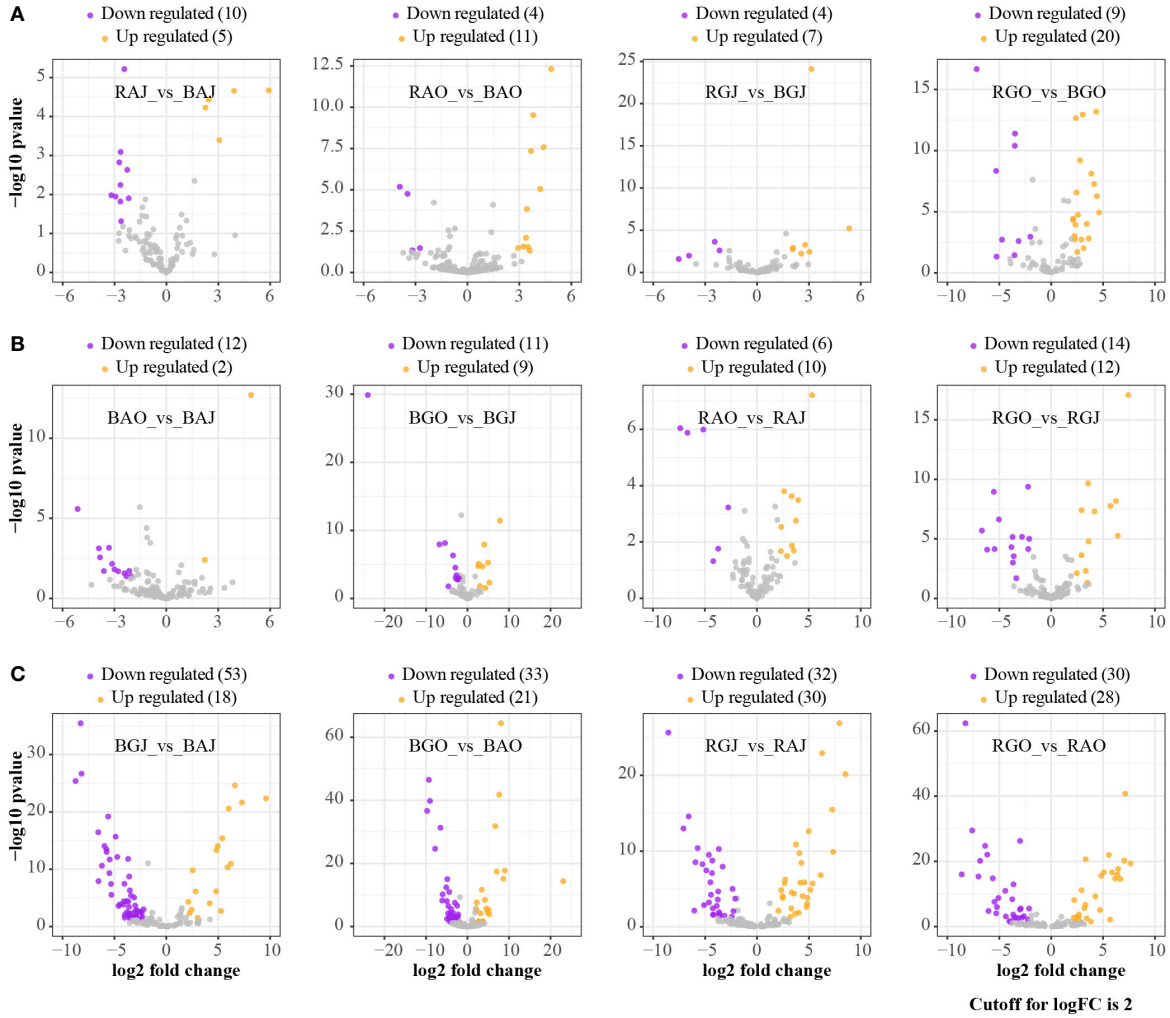
Differential expression analyses at family level in different compartments, seasons, and sites. **(A)** The ratio of rhizosphere to bulk. **(B)** The ratio of October to July. **(C)** The ratio of Genhe to Aershan. BAJ, bulk soil in Aershan, July; BAO, bulk soil in Aershan, October; BGJ, bulk soil in Genhe, July; BGO, bulk soil in Genhe, October; RAJ, rhizosphere soil in Aershan, July; RAO, rhizosphere soil in Aershan, October; RGJ, rhizosphere soil in Genhe, July; RGO, rhizosphere soil in Genhe, October.

More families were differentially expressed between sites (**Figure 3C**), while there were fewer families with differential expression in different compartments and seasons (**Figures 3A, B**). Clavulinaceae, Hoehnelomycetaceae, Hydnangiaceae, Hygrophoraceae, Pyronemataceae, Rhizopogonaceae, Sporidiobolaceae, and Tritirachiaceae were significantly express families in bulk soil. Mytilinidiaceae, Russulaceae, Trichosporonaceae, and Tulasnellaceae were significantly expressed in rhizosphere soil. In July, only Archaeorhizomycetaceae and Trimorphomycetaceae were significantly expressed. As for October, Clavulinaceae, Cordycipitaceae, Leucosporidiaceae, Mortierellaceae, Mycosphaerellaceae, Pezizaceae, Schizoporaceae, and Vibrisseaceae were significantly expressed. A total of 69 families showed differential expression between these two sites, with 40 families dominating at Aershan and 29 families dominating at Genhe (**Table 3**).

**Table 3:** The families of differential expression analyses in different compartments, seasons, and sites.

Part	Rhizosphere_vs_Bulk	October_vs_July	Genhe_vs_Aershan	
Down regulated	Clavulinaceae	Archaeorhizomycetaceae	Amphisphaeriaceae	Mrakiaceae
	Hoehnelomycetaceae	Trimorphomycetaceae	Atheliaceae	Nectriaceae
	Hydnangiaceae		Cephalothecaceae	Phaeosphaeriaceae
	Hygrophoraceae		Ceratobasidiaceae	Physalacriaceae
	Pyronemataceae		Cucurbitariaceae	Piskurozymaceae
	Rhizopogonaceae		Cystofilobasidiaceae	Psathyrellaceae
	Sporidiobolaceae		Dermateaceae	Pseudeurotiaceae
	Tritirachiaceae		Discinaceae	Pyronemataceae
			Fomitopsidaceae	Saccharomycetaceae
			Ganodermataceae	Sanchytriaceae
			Gomphidiaceae	Sebacinaceae
			Hyaloscyphaceae	Sordariaceae
			Hymenogastraceae	Sporidiobolaceae
			Hypocreaceae	Sporormiaceae
			Inocybaceae	Syzygosporaceae
			Leptosphaeriaceae	Thelephoraceae
			Leucosporidiaceae	Tremellaceae
			Lyophyllaceae	Tricholomataceae
			Melanommataceae	Trichomeriaceae
			Microascaceae	Trimorphomycetaceae
Up regulated	Mytilinidiaceae	Clavulinaceae	Archaeorhizomycetaceae	Hoehnelomycetaceae
	Russulaceae	Cordycipitaceae	Ascocorticiaceae	Hydnodontaceae
	Trichosporonaceae	Leucosporidiaceae	Aspergillaceae	Hygrophoraceae
	Tulasnellaceae	Mortierellaceae	Boletaceae	Leotiaceae
		Mycosphaerellaceae	Chrysozymaceae	Lipomycetaceae
		Pezizaceae	Clavariaceae	Mycosphaerellaceae
		Schizoporaceae	Clavicipitaceae	Myxotrichaceae
		Vibrisseaceae	Coniochaetaceae	Phacidiaceae
			Cordycipitaceae	Plectosphaerellaceae
			Cortinariaceae	Rhizopogonaceae
			Didymosphaeriaceae	Rutstroemiaceae
			Geminibasidiaceae	Serendipitaceae
			Gloniaceae	Tritirachiaceae
			Helotiaceae	Tulasnellaceae
			Herpotrichiellaceae	

These results indicated that there were significant differences in community composition and functional guilds between different compartments, seasons, and sites.

### 3.3 Fungal diversity and community assembly processes among different compartments, seasons, and sites

**Figure 4:**
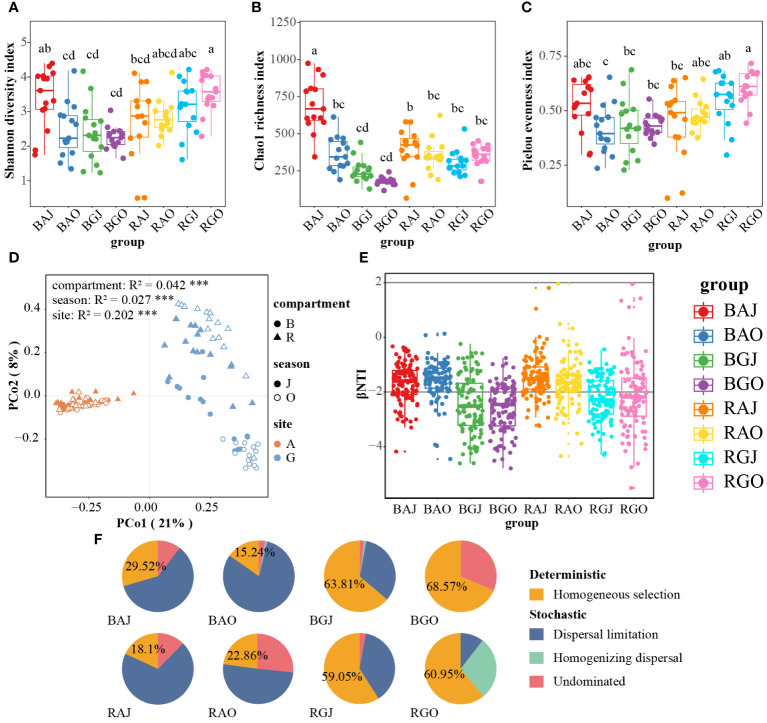
Differences in soil fungal communities among different compartments, seasons, and sites. The Shannon diversity index **(A)**, Chao1 richness index **(B)** and Pielou evenness index **(C)** of the fungal community. **(D)** Principal coordinate analysis (PCoA) based on the Bray-Curtis distance of fungal community. **(E)** The β-Nearest Taxon Index (βNTI) of the communities. **(F)** The proportion of deterministic and stochastic processes in fungal community assembly process in the two sites. Different lower case letters indicate significant difference among groups (P < 0.05). BAJ, bulk soil in Aershan, July; BAO, bulk soil in Aershan, October; BGJ, bulk soil in Genhe, July; BGO, bulk soil in Genhe, October; RAJ, rhizosphere soil in Aershan, July; RAO, rhizosphere soil in Aershan, October; RGJ, rhizosphere soil in Genhe, July; RGO, rhizosphere soil in Genhe, October.

The differences between the Shannon diversity, Chao1 richness, and Pielou evenness indices were significant in different groups, which consist of compartments, seasons, and sites. Shannon diversity and Pielou evenness indices of RGO were highest among these groups (**Figures 4A, C**). The group with the highest Chao1 richness index is BAJ (**Figures 4B**). Three-way ANOVA was used to analyze the effects of compartment, season, and site on alpha diversity. Among the single factors by Three-way ANOVA (**Table 4**), the compartment had a significant effect on the Shannon (F = 10.179, P = 0.002) and Pielou (F = 12.58, P = 0.001) indices, whereas both season (F = 24.934, P < 0.0001) and site (F = 85.252, P < 0.0001) had a significant effect on the Chao1 index.

**Table 4:** Effect of compartment, season, site and their interactions on fungal alpha diversity index by Three-way ANOVA.

Source of variation	diversity index	F	*P*
compartment	Chao1	0.896	0.346
	Shannon	10.179	**0.002**
	Pielou	12.58	**0.001**
season	Chao1	24.934	**<0.001**
	Shannon	1.11	0.294
	Pielou	0.085	0.771
site	Chao1	85.252	**<0.001**
	Shannon	0.014	0.906
	Pielou	3.384	0.068
compartment × season	Chao1	26.79	**<0.001**
	Shannon	8.96	**0.003**
	Pielou	5.994	**0.016**
compartment × site	Chao1	47.514	**<0.001**
	Shannon	19.627	**<0.001**
	Pielou	13.872	**<0.001**
season × site	Chao1	20.184	**<0.001**
	Shannon	4.329	**0.04**
	Pielou	2.807	0.097
compartment × season × site	Chao1	4.164	0.044
	Shannon	0.946	0.333
	Pielou	1.301	0.256

Bold values represent significant differences.

Principal coordinate analysis (PCoA) based on the Bray–Curtis distance (**Figure 4D**) indicated significant differences among multiple groups, especially between different sites (R² = 0.202, *P* = 0.001). The difference in fungal community structure between the rhizosphere and bulk soil in Genhe was relatively small, whereas the difference between the two seasons was significant. According to the results of the permutational multivariate analysis of variance (PERMANOVA), the grouping was credible (F = 9.088, *P* = 0.001).

According to the result of the β-nearest taxon index (βNTI, **Figure 4E**), the values were dispersed around −2. Homogeneous selection was more important than other processes in Genhe fungal community assembly, whereas dispersal limitation was more important than other processes in Aershan (**Figure 4F**). Soil fungal communities in Aershan were dominated by stochastic processes with average relative importance of 70.48−84.76%, whereas those in Genhe were dominated by deterministic processes with average relative importance of 59.05−68.57%. The proportions of deterministic and stochastic processes in the fungal community assembly process significantly differed between the two sites.

According to the results, there were significant differences in alpha diversity and beta diversity of fungal communities in the soil of *L. gmelinii*. Differently dominant processes in the community assembly may cause the above results.

### 3.4 The relationship between fungal community and environmental factors in bulk soil

We performed mantle tests on the relationship between individual environmental factors and the fungal community structure of bulk soil and found that all soil properties, temperature, and precipitation significantly affected the fungal community structure (**Table 5**). Among the environmental factors, TN (r = 0.734, *P* = 0.001) and pH (r = 0.174, *P* = 0.001) had the greatest effect on fungal community structure. Altogether, the correlation between the total environmental factors and community structure was not high (r = 0.174, *P* = 0.001).

**Table 5:** The Spearman’s correlations (r) between single environmental factor (Euclidean distance) and fungal community structure (Bray-Curtis distance) of the bulk soil determined by the Mantel test.

Factor	TN	SOC	AP	pH	CEC	Tmax	Tmin	Prec	Total
r	0.734	0.161	0.125	0.616	0.332	0.230	0.230	0.234	0.174
*P*	**0.001**	**0.003**	**0.001**	**0.001**	**0.001**	**0.001**	**0.001**	**0.001**	**0.001**

Bold values represent significant differences. TN, total nitrogen; SOC, soil organic carbon; AP, available phosphorus; CEC: cation exchange capacity; Tmax, the monthly minimum temperature; Tmin, the monthly minimum temperature; Prec: mean monthly precipitation.

**Figure 5:**
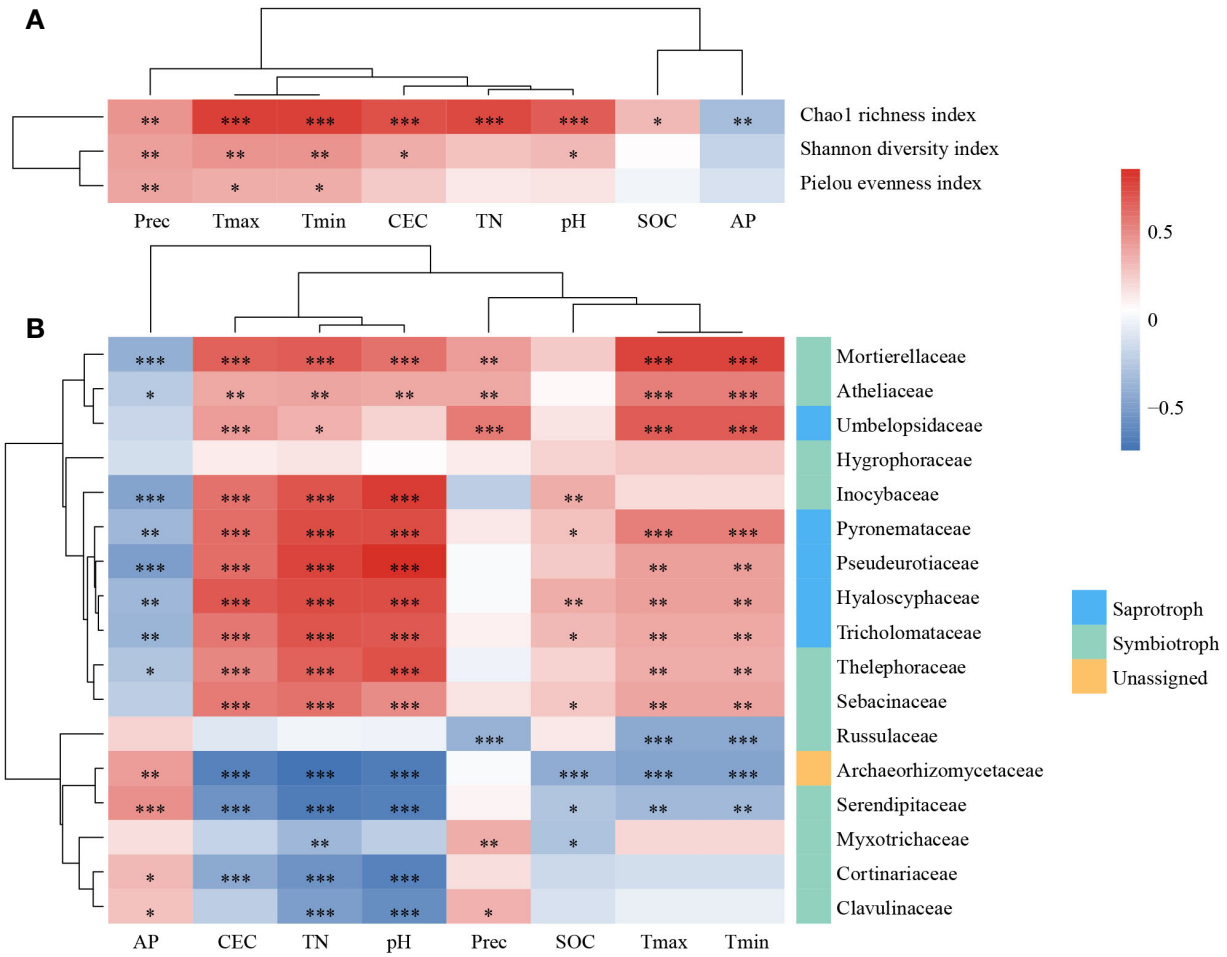
The impact of environmental factors on fungal communities. The Spearman’s correlation relationships between alpha diversity indices **(A)**, the relative abundance of dominate families **(B)** and environmental factors. *, *P* < 0.05; **, *P* < 0.01; ***, *P* < 0.001. TN, total nitrogen; SOC, soil organic carbon; AP, available phosphorus; CEC: cation exchange capacity; Tmax, the monthly minimum temperature; Tmin, the monthly minimum temperature; Prec: mean monthly precipitation.

In the correlation relationships between alpha diversity indices and environmental factors (**Figure 5A**), climatic factors had a significant effect on all three alpha diversity indices, and alpha diversity increased as temperature and precipitation increased. Additionally, the Chao1 evenness index was significantly influenced by the CEC, TN, pH, SOC, and AP. The effects of the CEC and pH on the Shannon diversity index were also significant.

To determine which taxa dominate the variation in fungal community structure in *L. gmelinii* forests under environmental changes, we evaluated the relationship between fungal families and the environment. In total, 16 of 17 dominant families were significantly correlated with environmental factors (**Figure 5B**), whereas the correlation between Hygrophoraceae and environmental factors was low and not significant. Russulaceae was significantly and negatively correlated with climatic factors. Inocybaceae were only significantly correlated with soil physicochemical properties. Mortierellaceae, Atheliaceae, Pyronemataceae, Pseudeurotiaceae, Hyaloscyphaceae, Tricholomataceae, and Thelephoraceae significantly correlated with AP, CEC, TN, pH, Tmax, and Tmin. A few of them were significantly correlated with Prec or SOC. Umbelopsidaceae was significantly and positively correlated with CEC, TN, and climatic factors. Archaeorhizomycetaceae and Serendipi,taceae were significantly correlated with environmental factors except Prec. Myxotrichaceae, Cortinariaceae, and Clavulinaceae were correlated with some soil physicochemical properties and Prec. The 17 dominant families were divided into two categories based on their correlations as a result of clustering.

Our results demonstrated that soil physicochemical properties and climatic factors played key roles in the diversity, composition and community structure of soil fungi.

## 4 Discussion

### 4.1 The fungal community of rhizosphere soil

Hosts produce exudates to regulate the rhizosphere microenvironment and provide carbon to microorganisms (Zhuang et al., 2020). Fungi combined with host roots to assist plants in absorbing carbon, nitrogen, phosphorus, and nutrients and play an important role in plant growth, development (Lu et al., 2018), and health (Berendsen et al., 2012). Many factors affect rhizosphere fungal communities, including vegetation type (Philippot et al., 2013), soil properties (Li et al., 2016), and biotic interactions (Paungfoo-Lonhienne et al., 2015). In our study, the fluctuations in Shannon and Chao1 indices were smaller in rhizosphere soil than in bulk soil. We supposed that the composition of fungal community in the rhizosphere was more stable. The alpha diversity of the fungal community in rhizosphere of *L. gmelinii* forest in Genhe was higher than that in bulk soil. However, the result was completely opposite in the soybean soil (Zhang et al., 2018). The selective effects of the root on specific taxa caused all the differences (Philippot et al., 2013). There were four families enriched in the rhizosphere soil of *L. gmelinii*. Russulaceae and Tulasnellaceae formed obligate mutualistic root symbionts with *L. gmelinii*, and they were also widely distributed in the mycorrhizae of orchid species (Li et al., 2021). Mytilinidiaceae and Trichosporonaceae were mainly involved in soil saprophytic processes and animal pathogenic transmission (Lourenço et al., 2020; Eagar et al., 2023). The families enriched in rhizosphere suggesting that those fungi were active in assisting nutrient uptake and transforming organic matter for *L. gmelinii*. Our results further confirmed that the host root system played a decisive role in soil fungi, and selected the specific fungal species. Exploring the status of rhizosphere fungal communities helps us understand the root health status of plants.

### 4.2 The effect of seasonal changes on soil fungal communities

Seasonal changes in temperature and precipitation have important effects on aboveground and underground organisms. A previous study has shown that fungal communities have better adaptability and resistance to cold environments compared with bacterial communities (Buckeridge et al., 2013). Regardless of seasonal changes, Basidiomycetes and Ascomycetes are the main components of fungal communities (Xie and Yin, 2022). However, Zeng et al. (2020) reported that the relative abundance of Ascomycota decreases as the annual average precipitation increases. Previous studies have shown that saprophytic fungi dominate the soil during the cold season, whereas ectomycorrhizal fungi have a greater advantage during the growing season (Voříšková et al., 2014; Santalahti et al., 2016; Zeng et al., 2022). At our sampling sites, temperature and precipitation were concentrated in July; the temperature in October was below zero and the soil was relatively dry. High soil moisture promotes the decomposition of dead leaves and increases soil nutrients, thereby affecting the functional groups of the soil fungal community (Brockett et al., 2012). During the drier season, soil fungal abundance decreases compared to the wet season, but fungal diversity increases (Kivlin and Hawkes, 2016). In our previous study, variations in temperature and precipitation were important factors influencing seasonal changes in the fungal communities (Zhao et al., 2023). In fact, seasonal changes were masked by sites in this study.

### 4.3 The effect of community assembly on fungal community

Deterministic and stochastic processes were used to determine soil fungal community structure. Selection, drift, speciation, and dispersal are usually responsible for the deterministic fitness differences, species abundance, creates new species, and movement by the four processes, among which selection is a deterministic process and diffusion is a stochastic process (Vellend, 2010). The fungal community assembly in Aershan was dominated by stochastic processes, whereas it was dominated by deterministic processes in Genhe. The body size is crucial for dispersal (Luan et al., 2020), it is also an important stochastic factor that leads to the low similarity of the two communities in our study. The assembly process of different communities can cause differences in the community structure. Nevertheless, the role of stochastic processes in microbial community assembly remains unclear (Nemergut et al., 2013; Gao et al., 2020; Jiao et al., 2020). Combining niche breadth, deterministic processes have a strong impact on specialist species with narrow niche breadth, while the generalist species with wide niche breadth has stronger competitiveness (Graham et al., 2016). In general, the community dominated by stochastic process has more resources around it and less competitive pressure (Jiao et al., 2020). Based on this understanding of community assembly, we consider that the soil condition of Genhe was more limited, resulting in contemporary selection dominating the fungal communities. The soil conditions in Aershan were better, providing more opportunities for fungal communities to drift, diversify, and disperse.

### 4.4 The effect of contemporary environment heterogeneity and historical contingencies on the structure of fungal communities

Notably, contemporary environmental heterogeneity and historical events (geographic distance) were the two major factors affecting the biographical distribution of soil fungal communities (Ramette and Tiedje, 2007). The two sampling sites for *L. gmelinii* were 460 km apart, and significant differences were found in the soil physicochemical properties between the two sites, so we considered the effect of geographical distance. In previous studies, the geographical scale played an important role in fungal community structure (Tedersoo et al., 2014; Zhang et al., 2018; Adamo et al., 2021). He et al. (2017) even suggested that spatial heterogeneity has a more significant effect than temporal consistency.

In the biogeographical distribution of soil fungi, the contemporary environment played a more important role (Zeng et al., 2019). As important soil nutrient factors, C, N, and P not only have a profound effect on aboveground vegetation but also provide an environment for the living dynamics of soil fungi. Nutritional factors such as TN, AP, and SOC are important in the regulation of fungal communities (Lauber et al., 2008; He et al., 2016; Dang et al., 2017; Li et al., 2020; Luo et al., 2023). Long-term nitrogen enrichment decreases the relative abundance and diversity of ectomycorrhizal taxa; however, the saprotrophs respond positively to nitrogen enrichment. (Zhou et al., 2016; Morrison et al., 2016). Nitrogen deposition affects the soil fungal community in *L. gmelinii* forests annually and is a major cause of global environmental change (Galloway et al., 2008). SOC significantly affects the structure of soil fungal communities (Sul et al., 2013; Li et al., 2017). A few families in our study were significantly related to SOC; however, all related families belonged to saprophytic and symbiotic fungi, such as ericoid mycorrhizal fungi, orchid mycorrhizal fungi, and ectomycorrhizal fungi. They degrade cellulose (Li et al., 2017), and assist hosts in obtaining carbon (Suetsugu et al., 2021). Nevertheless, when the other four physicochemical properties were significantly correlated with alpha diversity indices and some dominant families, AP showed a significant opposite correlation (**Figure 5**). Similar to our results for paddy fields in subtropical China, fungal richness and diversity indices declined significantly with increasing phosphorus rates (Liu et al., 2018). Bao et al. (2013) also found that the relationship between available phosphorus and fungal biodiversity showed a hump trend, slowly increasing before peaking and rapidly decreasing thereafter. As a non-nutritional factor, pH has a significant effect on soil fungal communities (Tedersoo et al., 2020). Compared with bacteria, fungi have a wider pH adaptation range (Rousk et al., 2010), but the dominant fungal taxa in different soil pH environments differ (Zhang et al., 2016). In our study, the correlation between the most dominant families and pH was high and extremely significant. CEC also affects the structure of fungal communities (Docherty et al., 2015). Some symbiotic taxa and the family Archaeorhiziomyceae were negatively correlated with pH, CEC, TN, and SOC. The relationship between these fungi and their hosts allows them to gain advantages in poor and acidic soils (Rosling et al., 2011; Vohník et al., 2016; Zhang and Sun, 2021). In addition to the factors studied, soil moisture content (Docherty et al., 2015) and salinity (Cao et al., 2021) also play important roles.

There were close relationships between TN, SOC, AP, pH, CEC and community structure, with TN and pH having the greatest effect (**Table 5**). The soil total nitrogen of our samples ranged from 0.81 g/kg to 6.88 g/kg, and the pH values ranged from 3.71 to 6.23, which was consistent with the values in the study of Miyamoto et al. (2021). We did not conduct further experiments on its optimal gradient, but pH around 4.89 (4.89 ± 0.08) may be its superior pH by ANOVA. Combined with the results that soil total nitrogen was positively correlated with alpha diversity indices (including Shannon diversity index, Chao1 richness index and Pielou evenness index), we supposed that high soil total nitrogen is more advantageous for soil fungal communities. So appropriate nitrogen supplementation and soil pH regulation can maintain and regulate the health of *L. gmelinii* forest.

## 5 Conclusion

Our findings revealed that compared to compartment and season, the impact of site on the soil fungal community of *L. gmelinii* was more important. Firstly, geographical distance determined low community similarity in terms of the stochastic process of community assembly. Secondly, there were significant differences in soil physicochemical properties between the two sites, which belonged to deterministic process. Environmental factors significantly affected fungal community structure, with soil TN and pH being the most important influencing factors. The geographical distance and environmental differences between the two sites result in significant differences in the soil fungal community assembly process. Meanwhile, seasonal changes appeared insignificant. This further deepens our understanding of the soil fungi of *L. gmelinii* forest and grasps the important soil environment in which *L. gmelinii* lives, which helps us to maintain the health of *L. gmelinii*.

## Data availability statement

The datasets presented in this study can be found in online repositories. The names of the repository/repositories and accession number(s) can be found in the article/supplementary material.

## Author contributions

WZ: Data curation, Investigation, Methodology, Software, Visualization, Writing – original draft, Writing – review & editing. KH: Data curation, Investigation, Methodology, Software, Writing – review & editing. RM: Data curation, Investigation, Methodology, Writing – review & editing. JL: Data curation, Investigation, Methodology, Writing – review & editing. YS: Conceptualization, Supervision, Writing – original draft, Writing – review & editing. BC: Conceptualization, Funding acquisition, Investigation, Project administration, Supervision, Writing – original draft, Writing – review & editing.
